# Radiolabeling and Biological Evaluation of Novel ^99m^Tc-Nitrido and ^99m^Tc-Oxo Complexes with 4-Methoxy-_L_-Phenylalanine Dithiocarbamate for Tumor Imaging

**DOI:** 10.3390/pharmaceutics14102196

**Published:** 2022-10-15

**Authors:** Guangxing Yin, Qing Ruan, Yuhao Jiang, Junbo Zhang

**Affiliations:** Key Laboratory of Radiopharmaceuticals of Ministry of Education, NMPA (National Medical Products Administration) Key Laboratory for Research and Evaluation of Radiopharmaceuticals, College of Chemistry, Beijing Normal University, Beijing 100875, China

**Keywords:** ^99m^Tc, 4-methoxy-_L_-phenylalanine, tumor imaging, dithiocarbamate, SPECT

## Abstract

To develop novel radiolabeled amino acid tumor imaging agents, 4-methoxy-_L_-phenylalanine dithiocarbamate (MOPADTC) was synthesized successfully, and two kinds of ^99m^Tc-labeled complexes ([^99m^Tc]TcN-MOPADTC and [^99m^Tc]TcO-MOPADTC) with high radiochemical purities (RCP > 95%) were obtained. The in vitro stability and partition coefficient were determined, and the results show that both of these complexes have good in vitro stability; [^99m^Tc]TcO-MOPADTC is hydrophilic, while [^99m^Tc]TcN-MOPADTC is slightly lipophilic. The biodistribution of [^99m^Tc]TcN-MOPADTC and [^99m^Tc]TcO-MOPADTC in mice bearing S180 tumors shows that the tumor uptake and tumor/muscle ratio of [^99m^Tc]TcO-MOPADTC were higher than the tumor uptake and tumor/muscle ratio of [^99m^Tc]TcN-MOPADTC. In addition, the tumor retention of [^99m^Tc]TcO-MOPADTC is better than the tumor retention of [^99m^Tc]TcN-MOPADTC. A competitive inhibition assay was performed, and the results indicate that [^99m^Tc]TcO-MOPADTC may enter cells primarily via the _L_-alanine/_L_-serine/_L_-cysteine (ASC) system. Single-photon emission computed tomography (SPECT) imaging of [^99m^Tc]TcO-MOPADTC shows obvious accumulation in tumor sites, suggesting that [^99m^Tc]TcO-MOPADTC is a novel potential tumor-imaging agent.

## 1. Introduction

Glucose-based [^18^F]FDG ([^18^F]fluorodeoxayglucose) is a positron emission tomography (PET) imaging agent, and is the most commonly used clinically [1,2]. Although [^18^F]FDG has a high uptake of tumors, some limitations still exist, such as producing false-positive or false-negative results and low image contrast in some cancer diagnoses [3,4]. The high uptake of [^18^F]FDG is related to increased glycolysis in tumor cells [5]. Amino acid and protein metabolism are enhanced in certain cancer cells [6], and amino acids are regarded as the key components of proteins and have many other functions that are essential for life, such as cellular metabolic survival and division [7,8].

Amino acid transport mechanisms can be classified according to many factors, such as transport saturation, kinetic behavior, substrate specificity, dependence on Na^+^ and sensitivity to pH [9,10]. The mechanisms can be divided into system A, system N, system ASC and system L [11,12,13]. System A is responsible mainly for the transport of small aliphatic amino acids, and system N is related mainly to the transport of amino acids with N atoms on the sidechain, such as histidine. System ASC transports small neutral amino acid molecules, such as alanine, cysteine and serine, while system L prefers to transport neutral large amino acids [14].

**Figure 1:**
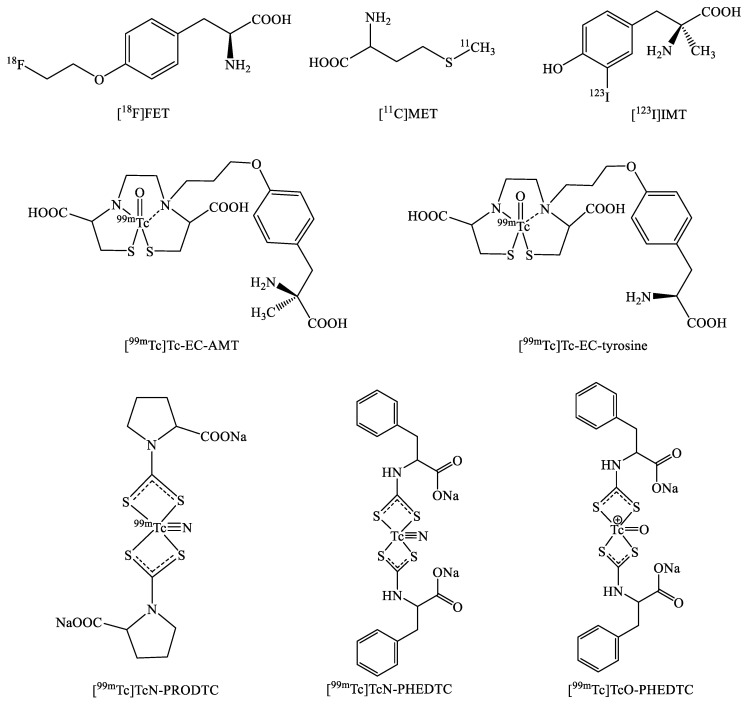
The proposed chemical structures of some mentioned radiolabeled amino acids.

Recently, aromatic amino acids have played a significant role in the clinical diagnostics of cancer. The chemical structures of some mentioned radiolabeled amino acids were shown in Figure 1. For example, [^18^F]FET (O-(2-[^18^F]fluoroethyl)-_L_-tyrosine) is a successful radiolabeled amino acid imaging agent that is transported through system L and has been employed in brain tumors, such as cerebral gliomas, with low uptake in normal brain tissue and high accumulation in brain cancer [15,16]. Biodistribution experiments confirmed that the uptake value of [^18^F]FET in inflammatory sites is lower than that of [^18^F]FDG and [^11^C]MET (_L_-[methyl-^11^C]methionine), indicating that [^18^F]FET could better distinguish a tumor from inflammation [17,18]. However, some limitations still exist, such as slow kidney clearance, the long retention time in the blood and the ineffectual detection rate in small tumors. The amino acid [^123^I]IMT (3-^123^I-iodo-a-methyl-_L_-tyrosine) is a kind of amino acid widely applied clinically as an imaging agent for glioma, small cell lung cancer and melanoma. It is transported into glioma cells mainly by system L. However, [^123^I]IMT is cleared rapidly, so that imaging must be performed within 45 min [19]. Tyrosine analogs with ^99m^Tc labels, such as [^99m^Tc]Tc-EC-AMT (EC: _L,L_-ethylenedicysteine, AMT: α-methyl tyrosine) and [^99m^Tc]Tc-EC-tyrosine, have attracted more attention, and the transport of both tracers involves system L [20]. However, the synthesis process of the ^99m^Tc-labeled compounds is complex, and the yield is low, so the clinical application is limited. Despite the substantial effort, the majority of the published radiotracers lack efficacy and are impractical for routine applications. Consequently, there is still plenty of room to fully explore the potential of amino-acid-based tracers as tumor imaging agents.

Technetium-99m is a γ-emitting radionuclide (E_γ_ = 140 keV, 89% abundance, T_1/2_ = 6.02 h) with a wide range of diagnostic applications in nuclear medicine. The ^99m^Tc has ideal nuclear properties for optimal imaging in SPECT. Additionally, ^99m^Tc is available in the form of pertechnetate ([^99m^Tc]TcO_4_^−^) with convenient production, as a ^99^Mo/^99m^Tc generator elutes in normal saline [21].

In our previous studies, some ^99m^Tc-labeled amino acid dithiocarbamates were prepared and evaluated, such as [^99m^Tc]TcN-PRODTC (PRODTC: proline dithiocarbamate), [^99m^Tc]TcN-PHEDTC and [^99m^Tc]TcO-PHEDTC (PHEDTC: phenylalanine dithiocarbamate) [22,23]. As previously reported, for [^99m^Tc]TcN-PRODTC and [^99m^Tc]TcN-PHEDTC, the uptake and retention of tumors was not satisfactory, although the synthesis procedures were easy and convenient. The tumor/blood ratio of [^99m^Tc]TcO-PHEDTC was low, and tumor retention was unsatisfactory [23].

In the development of ^99m^Tc radiopharmaceuticals, such as [^99m^Tc]Tc-MIBI (MIBI: 2-methoxy-2-methylpropylisonitrile) as a myocardial perfusion imaging agent and a tumor imaging agent, an interesting structural feature was that it had a methoxy group. The methoxy group may affect the biodistribution properties of the complex. In order to develop novel ^99m^Tc-labeled aromatic amino acids, 4-methoxy-_L_-phenylalanine (MOPA) was selected as the raw material to synthesize the corresponding dithiocarbamate ligand, and it was radiolabeled with [^99m^Tc][TcN]^2+^ and [^99m^Tc][TcO]^3+^ cores to obtain the corresponding ^99m^Tc-labeled complexes ([^99m^Tc]TcN-MOPADTC and [^99m^Tc]TcO-MOPADTC).

## 2. Materials and Methods

### 2.1. Reagents and Chemicals

All chemicals were purchased from commercial sources and used without further purification. Mass spectrum was recorded on a Triple TOF 5600 spectrometer (Concord, Canada). The NMR results were acquired on a 400 MHz JNM-ECS spectrophotometer (JEOL, Tokyo, Japan). Technetium-99m solution ([^99m^Tc]TcO_4_^−^) was eluted freshly from a ^99^Mo/^99m^Tc generator, which was purchased from ZHIBO Bio-Medical Tech, Beijing, China. Succinic dihydrazide (SDH) kits and glucoheptonate (GH) kits were obtained from Beijing Shihong Pharmaceutical Co., LTD (Bejing, China), Beijing Normal University, China. SPECT/CT imaging studies were performed on a SPECT/CT scanner, which was purchased from TriFoil imaging (Chatsworth, CA, USA).

### 2.2. Synthesis of MOPADTC

MOPADTC was prepared according to a previously reported method [23]. NaOH (0.413 g, 10 mmol) was added to water (20 mL) and stirred at room temperature. Then, 4-methoxy-_L_-phenylalanine (1.0 g, 5 mmol) was added to the above solution and stirred until it was completely dissolved. Carbon disulfide (3 mL, 50 mmol) was added to the above solution, and the reaction was performed at 0 °C for 4 h. The reaction progress was monitored through the pH value of the reaction solution. Upon the disappearance of NaOH, the pH of the reaction solution was approximately 7–8. Most of solvent and remaining carbon disulfide were removed under reduced pressure. Then, the residue was recrystallized by methanol, and was obtained as a white powder after drying in vacuo.

Regarding MOPADTC (0.90 g, yield, 57%), the ^1^H NMR results were as follows: ^1^H NMR (400 MHz, CD_3_OD) δ (ppm): 7.13 (t, *J* = 8.8 Hz, 2H), 6.82–6.72 (m, 2H), 3.72 (s, 3H), 3.45–3.36 (m, 1H), 3.02 (td, *J* = 13.2, 4.4 Hz, 1H), 2.71–2.66 (m, 1H). The ^13^C NMR results were as follows: ^13^C NMR (100 MHz, CD_3_OD) δ (ppm): 210.88, 176.53, 158.23, 130.65, 130.09, 113.06, 63.41, 54.34, 35.99. ESI-HRMS for C_11_H_11_NO_3_Na_2_S_2_ [M-Na]^−^: calcd. 292.0083, found 292.0081.

### 2.3. Radiolabeling and Quality Control Techniques

The [^99m^Tc]TcN-MOPADTC and [^99m^Tc]TcO-MOPADTC were prepared as follows: for preparing [^99m^Tc]TcN-MOPADTC, 1 mL of saline containing [^99m^Tc]TcO_4_^−^ (37–370 MBq) was added into a SDH kit, which consisted of 0.05 mg of stannous chloride, 5.0 mg of SDH and 5.0 mg of propylenediamine tetraacetic acid (PDTA). The mixture was reacted at room temperature for 20 min. Then, 1.0 mg of MOPADTC dissolved in 1.0 mL of saline was added to the mixture, and the reaction was performed for 20 min at 100 °C. For preparing [^99m^Tc]TcO-MOPADTC, 1 mL of saline containing [^99m^Tc]TcO_4_^−^ (37–370 MBq) was added to a GH kit, which consisted of 0.1 mg of stannous chloride and 20.0 mg of GH. The mixture was reacted at room temperature for 20 min. Then, 1.0 mg of MOPADTC dissolved in 1.0 mL of saline was added to the mixture, and the reaction was performed for 20 min at 100 °C.

The radiochemical purities (RCP) of both radiotracers were determined by thin-layer chromatography (TLC). TLC was performed using a polyamide strip as the solid phase and saline and acetonitrile as the eluents.

### 2.4. In Vitro Stability Study

The in vitro stability study of [^99m^Tc]TcN-MOPADTC and [^99m^Tc]TcO-MOPADTC was conducted according to the reported methods [23]. Briefly, the labeled complexes were kept at room temperature for 6 h. Then, the RCPs of the complexes were determined by TLC. In addition, 0.1 mL of radiolabeled complex was put into 0.1 mL of fresh mouse serum in a centrifuge tube, and the mixtures were incubated at 37 °C for 4 h. The in vitro serum stabilities were assessed by TLC.

### 2.5. Partition Coefficient (Log P)

The rate of dispersion of the radiolabeled complexes between the aqueous and organic phase, which is called partition coefficient, was measured. A total of 0.1 mL of the ^99m^Tc-complex was mixed with 1.0 mL of 1-octanol and 0.9 mL of phosphate buffer (PBS, 0.025 mol/L, pH 7.4). The mixtures were stirred vigorously, then centrifuged (3 min, 9000 rpm) so that the aqueous and organic phases were separated completely. For each phase, 0.1 mL of solution was removed, and the amount of radioactivity was measured in a γ-counter. The above experiments were repeated three times. The partition coefficient (P) was calculated as the radioactivity values of counts of the organic phases divided by those of the PBS. The final partition coefficient value was expressed as log P ± standard deviation (SD).

### 2.6. Biodistribution Studies

All biodistribution studies were performed in compliance with the national laws and regulations related to the conduct of animal experimentations. The S180 cell line was cultured in Kunming mice ascites. The S180 tumor models were established by injecting S180 cells (about 1 × 10^6^ in 0.1 mL of saline) into the left upper limb armpit of Kunming mice (female, 18–22 g) by subcutaneous injection. The tumors were allowed to grow for about one week, with diameters ranging from 5 to 8 mm. 

Then, 0.1 mL of ^99m^Tc complex (740 kBq/mL) was injected into Kunming female mice bearing S180 tumors via the tail vein. Mice were sacrificed in groups of five at different times post-injection. The tumors, blood, muscles and other organs of interest were collected, weighed and measured for radioactivity. The counting tubes, including a standard equivalent to 1% of the injected dose, were measured with a γ-counter. The results were calculated as the percent uptake of injected dose per gram of organ (%ID/g). The final results were expressed as the mean ± SD. 

### 2.7. Cellular Inhibition Studies of [^99m^Tc]TcO-MOPADTC

The murine sarcoma S180 cell line was used to characterize the transport mechanism of the radiotracers. The S180 tumor cells were obtained from the abdominal cavity of a sacrificed ascites mouse. The cells were centrifuged at 1000 rpm for 5 min, and the supernatant was discarded. The lower-layer cells were washed three times with saline. Then, the S180 cells were suspended in fresh Dulbecco’s modified eagle medium (DMEM) at a cell concentration of 5 × 10^6^ per mL for later use.

To characterize the transport mechanism of [^99m^Tc]TcO-MOPADTC, competitive inhibition studies were conducted with the S180 cell line according to a previously reported method [24,25,26]. A series of inhibitors was added to the cells in DMEM at a concentration of 20 mM. These inhibitors included selected amino acid transport inhibitors, such as 2-(methylamino)isobutyric acid hydrate (MeAIB, 40 mM, the specific inhibitor of system A) and 2-aminobicyclo-(2,2,1)-heptane-2-carboxylic acid (BCH, 40 mM, the specific inhibitor of system L). Additionally, natural amino acids were used as inhibitors, such as _L_-histidine (His, 40 mM, the inhibitor of system N) and a mixture of _L_-alanine/_L_-serine/_L_-cysteine (ASC, 13.3 mM of each amino acid, the inhibitor of system ASC). For each group (*n* = 5), 0.2 mL of cell solution, 0.5 mL (20 mM) of inhibitor, 0.1 mL of radiotracer and 0.2 mL of DMEM were added to the centrifuge tube successively. A total of 0.2 mL of cell solution, 0.7 mL of DMEM and 0.1 mL of radiotracer were added to the centrifuge tube as the control group. Then, they were incubated at 37 °C for 20 min. At the end of the incubation period, the samples were centrifuged at 9000 rpm for 5 min. Then, the supernatant was removed, and the residual sample with cells was washed with 0.5 mL of PBS twice. Finally, the residual sample with cells was counted with γ-counter. The results are expressed as the percentage of the cellular uptake in the control group.

### 2.8. SPECT/CT Imaging Studies

For the SPECT/CT imaging studies, Kunming female mice (18–22 g) bearing S180 tumors were injected with 0.1 mL of [^99m^Tc]TcO-MOPADTC (18 MBq) via the tail vein. SPECT/CT images were obtained from anesthetized mice using 1.5% isoflurane at 4 h post-injection. SPECT/CT images were analyzed using HISPECT software and VivoQuant 2.5 software.

## 3. Results

### 3.1. Synthesis of MOPADTC

**Scheme 1:**
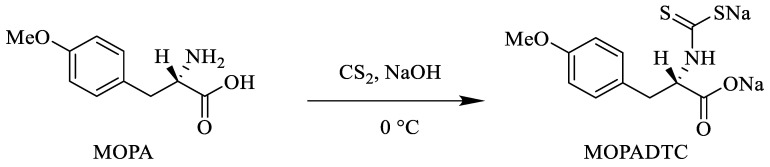
Synthesis route of the MOPADTC ligand.

The synthesis route is shown in Scheme 1. MOPADTC was successfully prepared by reacting MOPA with carbon disulfide (CS_2_) under basic conditions in 57% yield. It was characterized by ^1^H NMR, ^13^C NMR, mass spectrometry (MS). 

### 3.2. Radiolabeling and Quality Control 

**Scheme 2:**
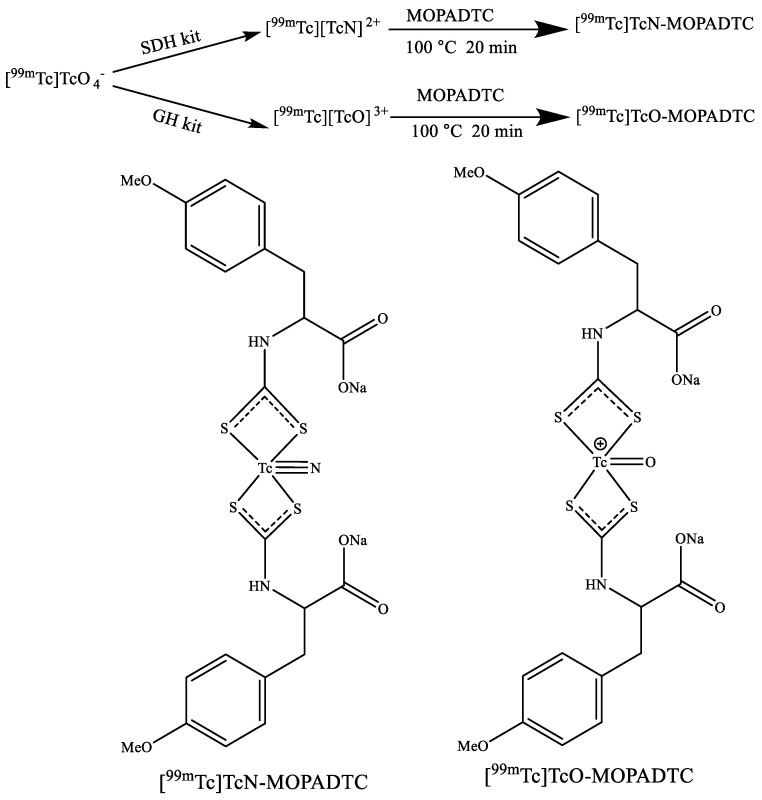
Radiosynthesis and proposed structures of [^99m^Tc]TcN-MOPADTC and [^99m^Tc]TcO-MOPADTC.

The radiosynthesis procedures and proposed structures of [^99m^Tc]TcN-MOPADTC and [^99m^Tc]TcO-MOPADTC are shown in Scheme 2. There is no corresponding rhenium reference of [^99m^Tc]TcN-MOPADTC and [^99m^Tc]TcO-MOPADTC, thus the proposed structure is an assumption based on reported literature [27,28,29]. Using kits, the preparations of the [^99m^Tc][TcN]^2+^ and [^99m^Tc][TcO]^3+^ intermediates were convenient and time-saving. Then, intermediates could undergo a ligand-exchange reaction with the MOPADTC ligand to obtain the final complexes with high RCP (>95%).

According to the reported literature [27,28,29], it can be presumed that [^99m^Tc]TcN-MOPADTC or [^99m^Tc]TcO-MOPADTC would form a structure with a Tc≡N bond or Tc=O bond linked to MOPADTC ligands through the four sulfur atoms. One dithiocarbamate ligand contains two sulfur atoms, whose coordination ability with technetium is better than that of nitrogen and oxygen atoms.

The radiochemical purities of the complexes were checked by TLC. For [^99m^Tc]TcN-MOPADTC in saline, [^99m^Tc]TcO_4_^−^and [^99m^Tc]TcN-MOPADTC migrated at the origin, but [^99m^Tc][TcN]^2+^ migrated at R_f_ = 0.8–1.0. In acetonitrile, [^99m^Tc]TcO_4_^−^migrated at R_f_ = 0.4–0.6, while [^99m^Tc]TcN-MOPADTC and [^99m^Tc][TcN]^2+^ remained at the origin. For [^99m^Tc]TcO-MOPADTC in saline, [^99m^Tc]TcO_4_^−^ and [^99m^Tc]TcO-MOPADTC migrated at the origin, but [^99m^Tc][TcO]^3+^ migrated at R_f_ = 0.8–1.0. In acetonitrile, [^99m^Tc]TcO_4_^−^ migrated at R_f_ = 0.4–0.6, while [^99m^Tc]TcO-MOPADTC and [^99m^Tc][TcO]^3+^remained at the origin.

### 3.3. Stability Experiments and Partition Coefficient

**Figure 2:**
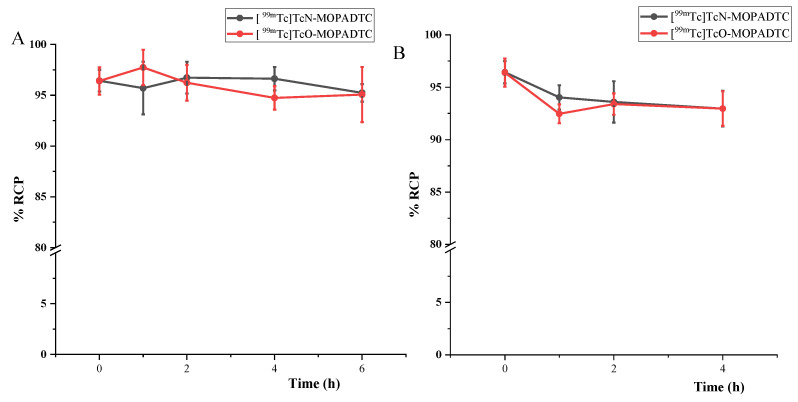
In vitro stability of [^99m^Tc]TcN-MOPADTC and [^99m^Tc]TcO-MOPADTC in saline (**A**) and mouse serum (**B**) (*n* = 3).

The in vitro stabilities of [^99m^Tc]TcN-MOPADTC and [^99m^Tc]TcO-MOPADTC were assayed by measuring their radiochemical purities (RCP). As shown in Figure 2, the RCP of both complexes was more than 90% after incubation either in saline at room temperature for 6 h or in mouse serum at 37 °C for 4 h, indicating good in vitro stabilities of the ^99m^Tc-labeled complexes. The partition coefficient values of [^99m^Tc]TcN-MOPADTC and [^99m^Tc]TcO-MOPADTC were 0.06 ± 0.06 and −0.37 ± 0.07, suggesting that [^99m^Tc]TcO-MOPADTC is hydrophilic while [^99m^Tc]TcN-MOPADTC is slightly lipophilic.

### 3.4. Biodistribution Studies

The results of the biodistribution studies of [^99m^Tc]TcN-MOPADTC and [^99m^Tc]TcO-MOPADTC are shown in Table 1. The uptake value in tumors at 0.5 h of [^99m^Tc]TcO-MOPADTC (1.18 ± 0.24 %ID/g) was twice as high as the uptake value in tumors of [^99m^Tc]TcN-MOPADTC (0.67 ± 0.12 %ID/g), while the tumor accumulation at 2 h of [^99m^Tc]TcO-MOPADTC (1.59 ± 0.45 %ID/g) was three times higher than the tumor accumulation of [^99m^Tc]TcN-MOPADTC (0.51 ± 0.05 %ID/g). Their tumor/blood ratios were close because the uptake of tumors and blood by [^99m^Tc]TcO-MOPADTC was higher than the uptake of tumors and blood of [^99m^Tc]TcN-MOPADTC. Additionally, the accumulation of muscle of [^99m^Tc]TcO-MOPADTC was lower, and the tumor uptake was higher, so that the tumor/muscle ratio of [^99m^Tc]TcO-MOPADTC was higher than the tumor/muscle ratio of [^99m^Tc]TcN-MOPADTC. The results suggest that [^99m^Tc]TcO-MOPADTC has the potential to be a novel ^99m^Tc-labeled tumor-imaging agent. Thus, the biodistribution results of [^99m^Tc]TcO-MOPADTC are discussed in detail in the following section.

As shown in Table 1, the tumor uptake of [^99m^Tc]TcO-MOPADTC increased with time in the first 2 h and was maintained at a constant value in the following 2 h, indicating that the tumor retention of [^99m^Tc]TcO-MOPADTC is excellent. At each time point, the accumulation in muscle was relatively low and diminished from 0.63 ± 0.14 %ID/g to 0.44 ± 0.11 %ID/g and 0.34 ± 0.12 %ID/g at 0.5 h, 2 h and 4 h, respectively. The initial uptake values of the heart, lung and stomach were not high, and most radioactivity was cleared after 4 h. The uptake doses of the liver and kidney remained high, indicating that the labeled complexes are metabolized and excreted mainly through the liver and kidney. 

### 3.5. Cellular Inhibition Studies of [^99m^Tc]TcO-MOPADTC

Based on the results of the biodistribution studies, the tumor uptake and tumor/muscle ratios of [^99m^Tc]TcO-MOPADTC were higher than those of [^99m^Tc]TcN-MOPADTC. Thus, [^99m^Tc]TcO-MOPADTC was chosen for further cellular studies by S180 cells to characterize the mechanisms of amino acid transport.

**Figure 3:**
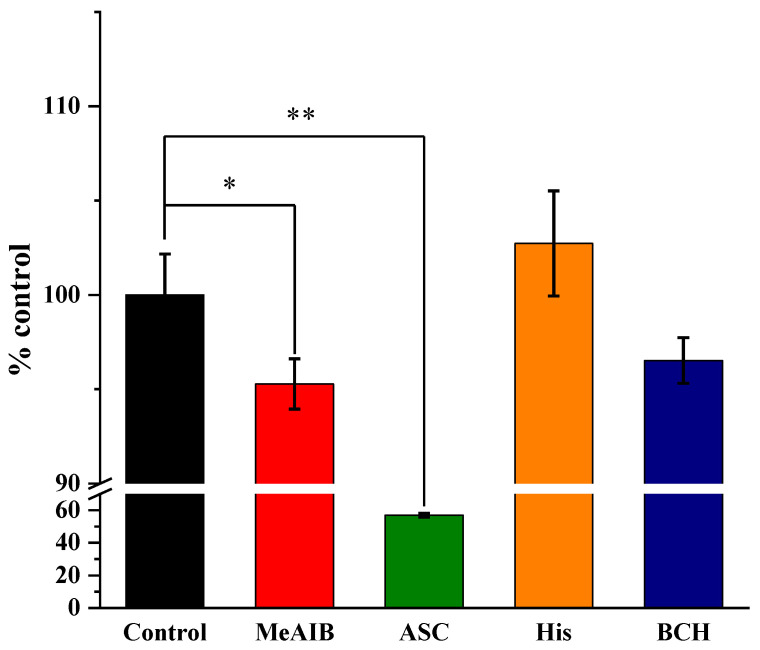
In vitro cellular uptake inhibition results of [^99m^Tc]TcO-MOPADTC conducted in S180 cells, incubation 20 min (* *p* < 0.05, ** *p* < 0.01).

To investigate the transport mechanisms of [^99m^Tc]TcO-MOPADTC, a variety of natural amino acids or amino acid transporter inhibitors were used to determine which amino acid transport systems were responsible for the uptake of [^99m^Tc]TcO-MOPADTC. As shown in Figure 3, the uptake of [^99m^Tc]TcO-MOPADTC was inhibited in S180 cells under ASC conditions (a decrease of 43%, *p* < 0.01) mainly and in MeAIB conditions (a decrease of 5%, *p* < 0.05) slightly. The results indicate that [^99m^Tc]TcO-MOPADTC may be transported mainly through system ASC and slightly through system A.

### 3.6. SPECT/CT Imaging

**Figure 4:**
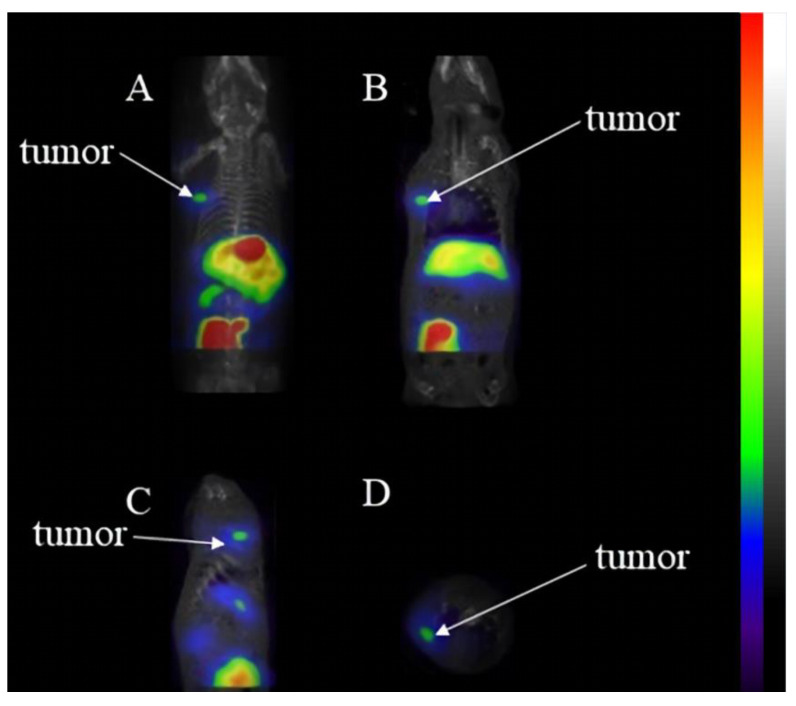
SPECT/CT images of [^99m^Tc]TcO-MOPADTC in mice bearing S180 cells at 4 h post-injection, visual imaging (**A**), coronal section (**B**), sagittal section (**C**) and transverse section (**D**).

As [^99m^Tc]TcO-MOPADTC had a higher tumor uptake and tumor/muscle ratio than [^99m^Tc]TcN-MOPADTC, [^99m^Tc]TcO-MOPADTC was selected as a promising agent for further SPECT/CT imaging studies. The SPECT/CT imaging results of [^99m^Tc]TcO-MOPADTC are shown in Figure 4, which were consistent with the biodistribution results. The tumor uptake was clearly observable. Additionally, the accumulations of the liver and kidney were obviously detectable from the imaging at 4 h post-injection.

## 4. Discussion

Currently, aromatic amino-acid-based radiopharmaceuticals play an important role in the field of oncologic imaging. In addition, ^99m^Tc is an ideal radionuclide with suitable energy and a suitable half-life for SPECT/CT. The ^99m^Tc-labeled radiotracer can be prepared in the kit form, which is convenient and simple for application. Moreover, SPECT scanners are much more numerous worldwide than PET scanners [30]. Furthermore, the diagnosis efficacy of cadmium zinc telluride (CTZ) SPECT will be nearly consistent with those of PET [31]. Consequently, it is necessary to develop novel ^99m^Tc-labeled aromatic amino-acid-based radiopharmaceuticals as tumor imaging agents for SPECT/CT. 

More importantly, chelators are required to conjugate the amino acid derivatives with ^99m^Tc [6,32]. Due to the fact that the coordination ability with ^99m^Tc of sulfur atoms is better those that of nitrogen and oxygen atoms, MOPADTC containing two sulfur atoms can make efficiently stable binding with [^99m^Tc][TcN]^2+^core and [^99m^Tc][TcO]^3+^ core to produce radiotracers [^99m^Tc]TcN-MOPADTC and [^99m^Tc]TcO-MOPADTC. 

**Figure 5:**
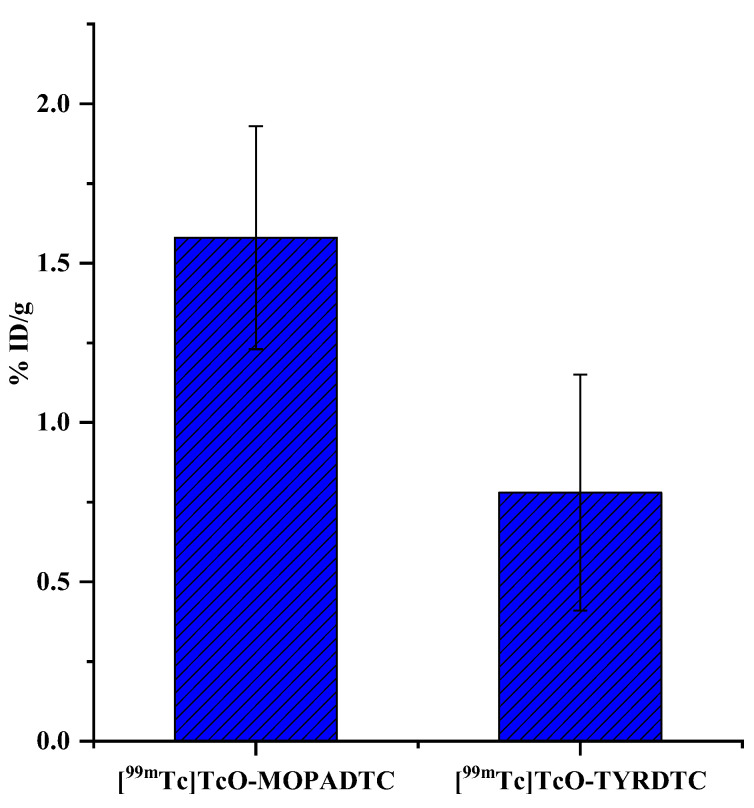
Comparison in tumor uptake at 4 h post-injection (*n* = 5) of [^99m^Tc]TcO-MOPADTC and [^99m^Tc]TcO-TYRDTC.

To verify whether the addition of a methoxy group on the benzene ring has an effect on the uptake of tumors, biodistribution studies of [^99m^Tc]TcO-TYRDTC (tyrosine dithiocarbamate was abbreviated to TYRDTC) at 4 h post-injection were also performed (*n* = 5) (the synthesis of TYRDTC (Scheme S1), radiolabeling (Scheme S2) and biodistribution results (Table S1) of [^99m^Tc]TcO-TYRDTC are provided in Supplementary data). As shown in Figure 5, the tumor uptake of [^99m^Tc]TcO-MOPADTC (1.58 ± 0.35 %ID/g) was higher than the tumor uptake of [^99m^Tc]TcO-TYRDTC (0.78 ± 0.37 %ID/g). The main structural difference among the ^99m^Tc-oxo complexes was that the group at the para-position of the benzene ring was different: the para-position of MOPA was methoxy, and the groups of tyrosine were phenolic hydroxyl respectively. [^99m^Tc]TcO-MOPADTC should be more lipophilic than [^99m^Tc]TcO-TYRDTC, thus making it easier for the former to penetrate the lipophilic membrane of tumor cells.

By comparing the biodistribution data of the new tracers, it can be concluded that the radioactivity accumulation was similar in the majority of organs, except for the kidneys. The kidney uptake of [^99m^Tc]TcO-MOPADTC was remarkably higher than the kidney uptake of [^99m^Tc]TcN-MOPADTC, which may be related to the hydrophilicity of the ^99m^Tc-oxo complex, while in the majority of other normal organs the uptake was similar. The tumor uptake of [^99m^Tc]TcO-MOPADTC was higher than that of [^99m^Tc]TcN-MOPADTC, and the tumor retention of [^99m^Tc]TcO-MOPADTC was more satisfactory. The result may be related to the positive charge of [^99m^Tc]TcO-MOPADTC. Due to the fact that the membrane potentials of tumor cells were negative, a cationic tracer, such as [^99m^Tc]TcO-MOPADTC, is electrophoretically driven through the transmembrane, and tends to accumulate in tumor cells. 

Currently, most amino-acid-based tracers are transported into cells via system A, ASC or L. Anti-1-amino-2-[^18^F]-fluorocyclobutane-1-carboxylic acid (anti-3-[^18^F]FACBC) is a radiolabeled probe used for its utility in the detection of recurrent prostate carcinoma and gliomas. The [^18^F]FACBC seems to enter cells through system ASC and system L [19]. Moreover, p-[^123^I]iodo-_L_-phenylalanine (IPA) and _L_-3-[^123^I]iodo-α-methyltyrosine (IMT) showed high accumulation in glioblastomas, and entered tumor cells through systems ASC and L[33]. By comparison, the inhibition studies in this study show that [^99m^Tc]TcO-MOPADTC is transported via system ASC mainly and via system A slightly in S180 tumor cells. We speculated that the conversion amino acid to amino acid dithiocarbamate, the addition of methoxy group and the different ^99m^Tc cores labeling may have an effect on the transport mechanisms of the tracer. Radiotracers targeting exact amino acid transport systems need to be further studied.

Due to its better biodistribution performance, [^99m^Tc]TcO-MOPADTC was chosen for further SPECT/CT imaging study. As predicted, [^99m^Tc]TcO-MOPADTC imaging exhibits evident tumor uptake. The uptakes of the kidneys and liver are also obvious, which indicates that the urinary and intestinal tracts are the major routes of excretion. The imaging results suggest that [^99m^Tc]TcO-MOPADTC has the potential to be used in molecular imaging to diagnose tumors.

## 5. Conclusions

In summary, MOPADTC was synthesized, and the corresponding radiolabeled complexes ([^99m^Tc]TcN-MOPADTC and [^99m^Tc]TcO-MOPADTC) were prepared with high radiochemical purity. The biodistribution and imaging studies in mice bearing S180 tumors show that [^99m^Tc]TcO-MOPADTC has a higher uptake of tumors and a higher tumor/muscle ratio than [^99m^Tc]TcN-MOPADTC. Compared with our previously synthesized dithiocarbamate tracer [^99m^Tc]TcO-PHEDTC [23], which shows a better tumor uptake and tumor/muscle ratio, the new tracer [^99m^Tc]TcO-MOPADTC shows better tumor retention and a higher tumor/blood ratio, characteristics that qualify it for diagnostic imaging. [^99m^Tc]TcO-MOPADTC is mainly transported via system ASC. It has the potential to be a novel ^99m^Tc-labeled tumor imaging agent.

## Figures and Tables

**Table 1:** Results of biodistribution of [^99m^Tc]TcN-MOPADTC and [^99m^Tc]TcO-MOPADTC (%ID/g ± SD, *n* = 5, T/B = tumor/blood ratio, T/M = tumor/muscle ratio).

Organ	[^99m^Tc]TcN-MOPADTC		[^99m^Tc]TcO-MOPADTC		
	0.5 h	2 h	0.5 h	2 h	4 h
heart	1.17 ± 0.28	0.98 ± 0.26	1.14 ± 0.11	0.81 ± 0.08	0.58 ± 0.27
liver	9.04 ± 1.59	4.63 ± 0.69	7.94 ± 0.87	4.62 ± 0.52	4.22 ± 1.29
lung	2.33 ± 0.63	2.11 ± 0.24	2.06 ± 0.31	1.64 ± 0.07	1.30 ± 0.26
kidney	7.79 ± 0.88	6.05 ± 1.10	9.14 ± 2.43	13.05 ± 1.97	9.88 ± 1.87
spleen	1.04 ± 0.39	0.75 ± 0.12	1.24 ± 0.35	0.80 ± 0.18	0.68 ± 0.19
stomach	1.25 ± 0.33	2.99 ± 0.95	2.22 ± 1.39	1.86 ± 0.82	0.79 ± 0.21
bone	0.45 ± 0.29	0.43 ± 0.13	1.02 ± 0.17	0.70 ± 0.07	0.68 ± 0.25
intestine	6.56 ± 0.93	4.07 ± 0.64	4.36 ± 0.95	2.92 ± 1.31	0.93 ± 0.25
muscle	0.41 ± 0.05	0.50 ± 0.10	0.63 ± 0.14	0.44 ± 0.11	0.34 ± 0.12
blood	1.19 ± 0.13	0.46 ± 0.06	3.36 ± 051	1.82 ± 0.35	2.07 ± 0.57
tumor	0.67 ± 0.12	0.51 ± 0.05	1.18 ± 0.24	1.59 ± 0.45	1.58 ± 0.35
T/B	0.56 ± 0.07	1.11 ± 0.25	0.35 ± 0.07	0.87 ± 0.07	0.76 ± 0.37
T/M	1.63 ± 0.59	1.02 ± 0.32	1.87 ± 0.31	3.61 ± 0.42	4.65 ± 1.06

## Supplementary Materials

The following supporting information can be downloaded at: https://www.mdpi.com/article/10.3390/pharmaceutics14102196/s1, Scheme S1. Synthesis route of the TYRDTC ligand. Scheme S2. Radiosynthesis and proposed structure of [^99m^Tc]TcO-TYRDTC. Table S1. Results of biodistribution of [^99m^Tc]TcO-TYRDTC at 4 h post-injection (%ID/g ± SD, n = 5, T/B = tumor/blood ratio, T/M = tumor/muscle ratio).

## References

[1] Hicks R.J. (2009). Role of 18F-FDG PET in assessment of response in non–small cell lung cancer. J. Nucl. Med..

[2] Boellaard R., Delgado-Bolton R., Oyen W.J.G., Giammarile F., Tatsch K., Eschner W., Verzijlbergen F.J., Barrington S.F., Pike L.C., Weber W.A. (2015). FDG PET/CT: EANM procedure guidelines for tumour imaging: Version 2.0. Eur. J. Nucl. Med. Mol. Imaging.

[3] Chang J.M., Lee H.J., Goo J.M., Lee H.-Y., Lee J.J., Chung J.-K., Im J.-G. (2006). False positive and false negative FDG-PET scans in various thoracic diseases. Korean J. Radiol..

[4] Rosenbaum S.J., Lind T., Antoch G., Bockisch A. (2006). false-positive FDG PET uptake−the role of PET/CT. Eur. Radiol..

[5] Williams S.-P., Flores-Mercado J.E., Port R.E., Bengtsson T. (2012). Quantitation of glucose uptake in tumors by dynamic FDG-PET has less glucose bias and lower variability when adjusted for partial saturation of glucose transport. EJNMMI Res..

[6] Mogadam H.Y., Erfani M., Nikpassand M., Mokhtary M. (2020). Preparation and assessment of a new radiotracer technetium-99m-6-hydrazinonicotinic acid-tyrosine as a targeting agent in tumor detecting through single photon emission tomography. Bioorgan. Chem..

[7] Huang C., McConathy J. (2013). Radiolabeled amino acids for oncologic imaging. J. Nucl. Med..

[8] Wu G. (2009). Amino acids: Metabolism, functions, and nutrition. Amino Acids.

[9] Hatzoglou M., Fernandez J., Yaman I., Closs E. (2004). Regulation of cationic amino acid transport: The story of the CAT-1 transporter. Annu. Rev. Nutr..

[10] Hyde R., Taylor P.M., Hundal H.S. (2003). Amino acid transporters: Roles in amino acid sensing and signalling in animal cells. Biochem. J..

[11] McConathy J., Yu W., Jarkas N., Seo W., Schuster D.M., Goodman M.M. (2012). Radiohalogenated nonnatural amino acids as PET and SPECT tumor imaging agents. Med. Res. Rev..

[12] McGIVAN J.D., Pastor-Anglada M. (1994). Regulatory and molecular aspects of mammalian amino acid transport. Biochem. J..

[13] Closs E.I., Boissel J.-P., Habermeier A., Rotmann A. (2006). Structure and function of cationic amino acid transporters (CATs). J. Membr. Biol..

[14] Fuchs B.C., Bode B.P. (2005). Amino acid transporters ASCT2 and LAT1 in cancer: Partners in crime?. Semin. Cancer Biol..

[15] Stegmayr C., Stoffels G., Filß C., Heinzel A., Lohmann P., Willuweit A., Ermert J., Coenen H.H., Mottaghy F.M., Galldiks N. (2021). Current trends in the use of O-(2-[18F]fluoroethyl)-L-tyrosine ([18F]FET) in neurooncology. Nucl. Med. Biol..

[16] Siddiq I.S., Atwa S.T., Shama S.A., Eltaoudy M.H., Omar W.M. (2018). Radiosynthesis and modified quality control of O-(2-[18F]fluoroethyl)-L-tyrosine ([18F]FET) for brain tumor imaging. Appl. Radiat. Isot..

[17] Kaim A.H., Weber B., Kurrer M.O., Westera G., Schweitzer A., Gottschalk J., Von Schulthess G.K., Buck A. (2002). 18F-FDG and 18F-FET uptake in experimental soft tissue infection. Eur. J. Nucl. Med. Mol. Imaging.

[18] Rau F.C., Weber W.A., Wester H.-J., Herz M., Becker I., Krüger A., Schwaiger M., Senekowitsch-Schmidtke R. (2002). O-(2-[18F]Fluoroethyl)-L-tyrosine (FET): A tracer for differentiation of tumour from inflammation in murine lymph nodes. Eur. J. Nucl. Med. Mol. Imaging.

[19] Zhu J., Song X., Zhang J. (2018). Development of amino acid-based radiopharmaceuticals for tumor imaging. MiniRev. Med. Chem..

[20] Kong F.-L., Zhang Y., Ali M.S., Oh C., Mendez R., Kohanim S., Tsao N., Chanda M., Huang W.-C., Yang D.J. (2010). Synthesis of 99mTc-EC-AMT as an imaging probe for amino acid transporter systems in breast cancer. Nucl. Med. Commun..

[21] Papagiannopoulou D. (2017). Technetium-99m radiochemistry for pharmaceutical applications. J. Label. Compd. Radiopharm..

[22] Liu M., Lin X., Song X., Cui Y., Li P., Wang X., Zhang J. (2013). Synthesis and biodistribution of a novel 99mTc nitrido radiopharmaceutical with proline dithiocarbamate as a potential tumor imaging agent. J. Radioanal. Nucl. Chem..

[23] Zhu J., Wang Y., Li Z., Fang S., Zhang J. (2014). Synthesis and biological evaluation of novel 99mTc-oxo and 99mTc-nitrido complexes with phenylalanine dithiocarbamate for tumor imaging. J. Radioanal. Nucl. Chem..

[24] Chen J., Li C., Hong H., Liu H., Wang C., Xu M., Han Y., Liu Z. (2019). Side chain optimization remarkably enhances the in vivo stability of 18F-labeled glutamine for tumor imaging. Mol. Pharmaceutics..

[25] Wu R., Liu S., Liu Y., Sun Y., Cheng X., Huang Y., Yang Z., Wu Z. (2019). Synthesis and biological evaluation of [18F](2S,4S)4-(3-fluoropropyl) arginine as a tumor imaging agent. Eur. J. Med. Chem..

[26] Bouhlel A., Zhou D., Li A., Yuan L., Rich K.M., McConathy J. (2015). Synthesis, radiolabeling, and biological evaluation of (R)- and (S)-2-amino-5-[18F]fluoro-2-methylpentanoic acid ((R)-, (S)-[18F]FAMPe) as potential positron emission tomography tracers for brain tumors. J. Med. Chem..

[27] Zhang S., Zhang W., Wang Y., Jin Z., Wang X., Zhang J., Zhang Y. (2011). Synthesis and biodistribution of a novel 99mTcN complex of norfloxacin dithiocarbamate as a potential agent for bacterial infection imaging. Bioconjug. Chem..

[28] Blower P.J., Singh J., Clarke S.E. (1991). The chemical identity of pentavalent technetium-99m-dimercaptosuccinic acid. J. Nucl. Med..

[29] Zhang J., Yu Q., Huo J., Pang Y., Yang S., He Y., Tang T., Yang C., Wang X. (2010). Synthesis and biodistribution of a novel 99mTc-DMSA-metronidazole ester as a potential tumor hypoxia imaging agent. J. Radioanal. Nucl. Chem..

[30] Wyngaert T.V.D., Elvas F., De Schepper S., Kennedy J.A., Israel O. (2020). SPECT/CT: Standing on the shoulders of giants, it is time to reach for the sky!. J. Nucl. Med..

[31] de Souza A.C.D.A., Harms H.J., Martell L., Bibbo C., Harrington M., Sullivan K., Hainer J., Dorbala S., Blankstein R., Taqueti V.R. (2022). Accuracy and reproducibility of myocardial blood flow quantification by single photon emission computed tomography imaging in patients with known or suspected coronary artery disease. Circ. Cardiovasc. Imaging.

[32] Maresca K.P., Marquis J.C., Hillier S.M., Lu G., Femia F.J., Zimmerman C.N., Eckelman W.C., Joyal J.L., Babich J.W. (2010). Novel polar single amino acid chelates for technetium-99m tricarbonyl-based radiopharmaceuticals with enhanced renal clearance: Application to octreotide. Bioconjug. Chem..

[33] Samnick S., Richter S., Romeike B.F., Heimann A., Feiden W., Kempski O., Kirsch C.-M. (2000). Investigation of iodine-123-labelled amino acid derivatives for imaging cerebral gliomas: Uptake in human glioma cells and evaluation in stereotactically implanted C6 glioma rats. Eur. J. Nucl. Med..

