# Supplementary material for: Radiolabeling and Biological Evaluation of Novel 99mTc-Nitrido and 99mTc-Oxo Complexes with 4-Methoxy-L-Phenylalanine Dithiocarbamate for Tumor Imaging
Source: Pharmaceutics. 2022 Oct 15;14(10):2196. doi: 10.3390/pharmaceutics14102196 (PMC9607073; doi:10.3390/pharmaceutics14102196)
Supplement: Supplementary file 1 [file pharmaceutics-14-02196-s001.zip › pharmaceutics-1938177-supplementary.pdf]

Supplementary data: Radiolabeling and biological evaluation of novel ^{99m}Tc-nitrido and ^{99m}Tc-oxo complexes with 4-methoxy-L-phenylalanine dithiocarbamate for tumor imaging

Synthesis of TYRDTC

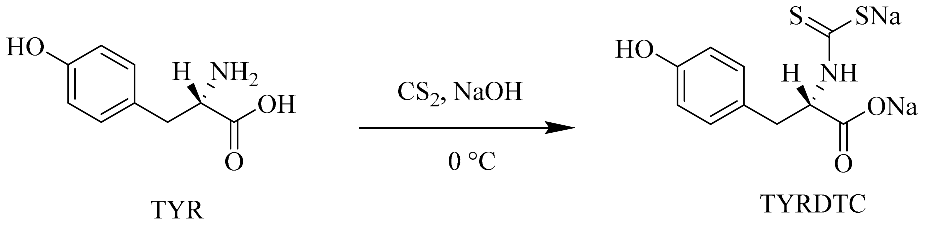

Scheme S1. Synthesis route of the TYRDTC ligand.

TYRDTC was prepared as follows: NaOH (0.62 g, 10 mmol) was added to water (5 mL) and THF (5 mL), then stirred at room temperature. Then, tyrosine (1.0 g, 5 mmol) was added to the above solution and stirred until it was completely dissolved. Carbon disulfide (3 mL, 50 mmol) was added to the above solution, and the reaction was performed at 0 °C for 4 h. Most of solvent and remaining carbon disulfide were removed under reduced pressure. The residue was recrystallized by methanol. TYRDTC (0.98 g, 53%) was obtained as white powder after drying *in vacuo*. ^{1}H NMR (400 MHz, CD_3OD) δ: 7.13 (d, J = 8.8 Hz, 2H), 6.62 (dt, 2H, J = 8.4, 2.4 Hz), 4.90 (t, 1H, J = 5.1 Hz), 3.32-3.28 (m, 2H). The ^{13}C NMR results were as follows: ^{13}C NMR (101 MHz, CD_3OD) δ:211.46, 176.98, 155.45, 131.02, 129.12, 114.65, 63.36, 36.27. ESI-MS for $C_{10}H_9NNa_2S_2O_3$ [M-H]$^-$: calcd. 300.98, found 300.17.

Radiolabeling and Biodistribution results of [^{99m}Tc]TcO-TYRDTC

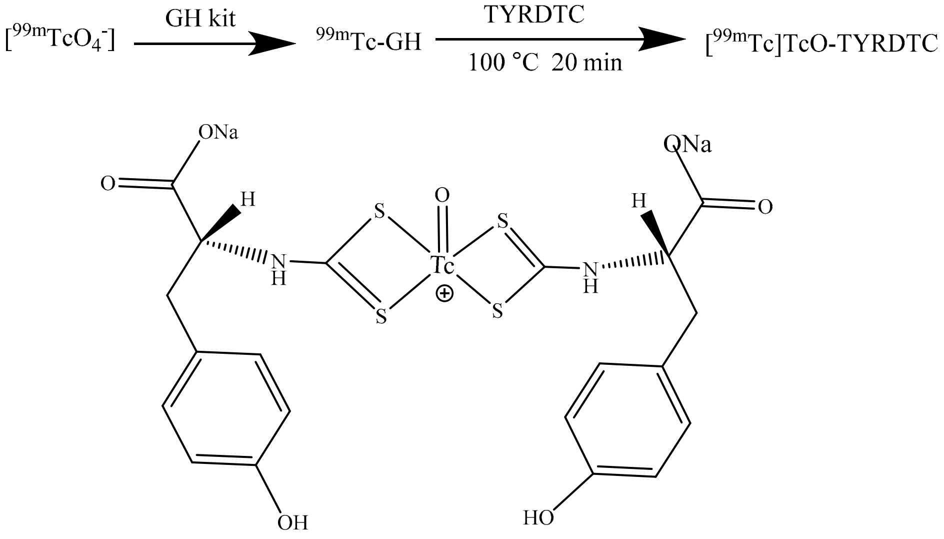

[^{99m}Tc]TcO-TYRDTC

Scheme S2. Radiosynthesis and proposed structure of [^{99m}Tc]TcO-TYRDTC.

For preparing [^{99m}Tc]TcO-TYRDTC, 1 mL of saline containing $^{99m}TcO_4^-$ (37-370 MBq) was added to a GH kit, which consisted of 0.1 mg of stannous chloride and 20.0 mg of GH. The mixture was reacted at room temperature for 20 min. Then, 1.0 mg of TYRDTC dissolved in 1.0 mL of saline was added to the mixture, and the reaction was performed for 20 min at 100 °C.

The radiochemical purity (RCP) of [^{99m}Tc]TcO-TYRDTC was determined by thin-layer chromatography (TLC). TLC was performed using a polyamide strip as the solid phase and saline and acetonitrile as the eluents. For [^{99m}Tc]TcO-TYRDTC in saline, $^{99m}TcO_4^-$ and [^{99m}Tc]TcO-TYRDTC migrated at the origin, but ^{99m}Tc-GH migrated at R_f = 0.8-1.0. In acetonitrile, $^{99m}TcO_4^-$ migrated at R_f = 0.4-0.6, while [^{99m}Tc]TcO-TYRDTC and ^{99m}Tc-GH remained at the origin.

Table S1. Results of biodistribution of [^{99m}Tc]TcO-TYRDTC at 4 h post-injection (%ID/g ± SD, n = 5, T/B = tumor/blood ratio, T/M = tumor/muscle ratio).

**organ**	%ID/g ± SD
heart	0.38 ± 0.10
liver	5.99 ± 0.68
lung	0.72 ± 0.24
kidney	6.37 ± 2.35
spleen	0.76 ± 0.24
stomach	1.24 ± 0.83
bone	0.34 ± 0.06
intestine	0.95 ± 0.43
muscle	0.17 ± 0.06
blood	0.97 ± 0.14
tumor	0.78 ± 0.37
T/B	0.80 ± 0.26
T/M	4.44 ± 1.19
